# The top 50 most cited articles in carpal tunnel syndrome research: A bibliometrics study

**DOI:** 10.1097/MD.0000000000028012

**Published:** 2022-01-07

**Authors:** Lei Lei, Shanshan Yin, Fanyuan Meng, Ya Zhou, Xuan Xu, Li Juan Ao, Rui Xu, Mo Xian Chen

**Affiliations:** a Department of Orthopaedics and Traumatology, Faculty of Medicine, The Chinese University of Hong Kong, Hong Kong, China; b Department of Neurology, Anhui Medical University, Hefei, Anhui, China; c School of Rehabilitation, Kunming Medical University, Kunming, China; d Rehabilitation Medicine Department, The First People's Hospital of Yunnan Province, China.

**Keywords:** bibliometrics, carpal tunnel syndrome, data analysis

## Abstract

**Background:**

Citation analysis was applied to identify the influential studies in the specific field. More and more literature related to carpal tunnel syndrome (CTS) have been published in recent years. To our knowledge, no one has performed a citation analysis of CTS. Thus, our study identified the top 50 influential articles pertaining to CTS and conduct an analysis of their characteristics.

**Methods:**

The Web of Science database was used to identify all the articles from 1900 to 2020. We obtained the top 50 articles ranked by citation times, and articles were included and excluded based on the relevance to CTS. Also, we collected the information about journal name, level of evidence, source country and institution, and research type for further analysis.

**Results:**

The top 50 articles were published between 1959 and 2012. The number of citations ranged from 151 to 1083. The citation density was between 3.23 and 40.27 per year. *Muscle Nerve* published most articles in CTS research, followed by *Journal of Bone and Joint Surgery American Volume*. The USA was the leading country, and all the top 5 institutions were from the USA. Katz JN with the highest *h-index* published most articles. Level III was the most common evidence level.

**Conclusions:**

We identified the top 50 cited articles related to CTS. These influential articles might provide researchers with a comprehensive list of the major contribution related to CTS research.

## 1 Introduction

Carpal tunnel syndrome (CTS), the most common entrapment neuropathy, is caused by median nerve compression at the wrists.^[1]^ The typical symptoms of CTS include pain, numbness, and tingling, mostly in the thumb, middle finger, and index finger.^[2]^ The prevalence of CTS is 3% in general population.^[3]^ Currently, the diagnostic criteria are clear, and we also have standard procedures for CTS. However, many details about CTS remain unknown, especially integrating theory with practice. We still explore convenient diagnostic options, like ultrasonography.^[4]^ Besides, novel strategies, such as shock wave therapy^[5]^ and platelet-rich plasma,^[6]^ have been applied to treat patients with CTS in recent years. It was attracting more and more researchers to focus on the research field, and many papers related to CTS were published.^[7,8]^ However, significant studies pertaining to CTS are often overlooked. It is imperative to conduct a citation analysis to identify the impact of publications in the specific field.

The number of citations is one of the ways to assess the academic influence of a published paper.^[9]^ Citation analysis is a bibliometric tool used to identify the influential studies in a given field.^[10]^ Besides, we use this tool to analyze the characteristics and qualities of these studies.^[11]^ These studies might have the potential to influence the direction of future research.^[12]^ In addition, citation analysis can identify the key journals, main countries and institutions, and core authors related to CTS research area.^[13]^ Several studies have been conducted to identify the top-cited articles in the orthopedic field in recent years, including rotator cuff research,^[14]^ total hip arthroplasty,^[11]^ knee arthroplasty,^[15]^ and osteoporosis.^[16]^ To our knowledge, no authors have analyzed the top-cited articles on CTS. Identification of the vital literature can provide researchers with an insight into the trends of this topic and might also guide a clinical decision. The current study aimed to provide the most popular topics, dominant study designs, and the core countries, institutions, journals, and authors in this field. Thus, we listed the 50 top-cited articles related to CTS to help researchers follow the most vital advances in this field.

## 2 Methods

We searched from the Web of Science (WoS) Core Collection to identify all the articles on December 28, 2020. The search term used was “carpal tunnel syndrome” under the “title” category. The retrieved time was between the earliest data available (1900) to 2020. The language of articles was limited to English. Besides, to improve retrieval accuracy, only articles and reviews were involved in our search. We ranked all the 50 top-cited articles by citation times, from highest to lowest. Two authors were assigned to independently screen the articles to identify the top 50 cited articles on CTS. Only the articles focused on CTS were included. The articles just peripherally mentioned CTS were excluded. All disagreements were resolved either through discussion or by a third author. The current study was not reviewed by an ethics committee because it is a bibliometric analysis of published articles, and no patients were included in this study.

We extracted the following information for all articles: first author, the title, year of publication, number of time-cited, citation density (the number of citations per year), journal name, level of evidence, source country and institution, and research type. Two independent authors identified the type of articles and level of evidence for clinical articles. The level of academic outputs of authors was assessed by *h-index*, which means the author have *h* papers have been cited at least *h* times.^[17]^ The methodology of evaluating the level of evidence of clinical trials was based on *The Journal of Bone & Joint Sugery*.^[18]^ We also obtained the journal impact factor (IF) in 2019 and partition from Journal Citation Reports (JCR).

## 3 Results

**Figure 1:**
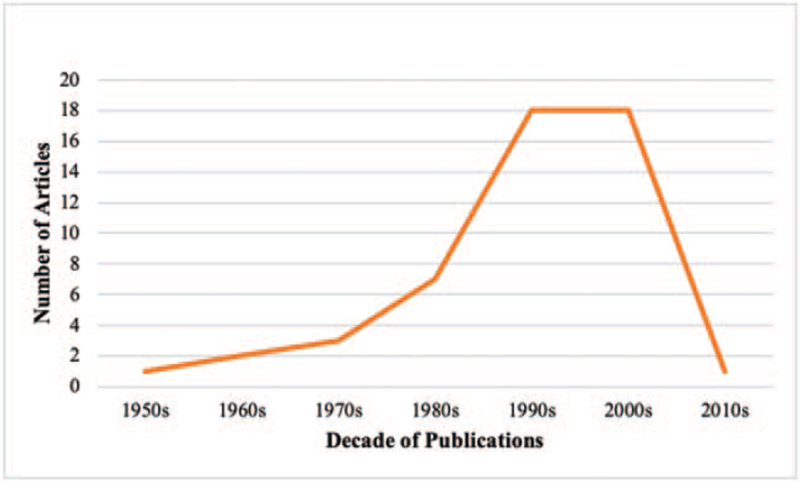
Number of articles by decade of publication.

We obtained the 50 top-cited articles related to CTS based on the WoS. Table 1 lists the basic information of the top 50 most cited articles. The number of citations ranged from 151 to 1083 (mean 270). The citation density was between 3.23 and 40.27 per year (mean 11). The top 50 articles that were published spanned from 1959 to 2012, with most of the articles published in the 1990s with the same number in the 2000s. Besides, the number of published articles was increased from the 1950s to the 1990s (Fig. 1).

**Table 1:** The basic information of top 50 cited articles.

Rank	First-author	Title	Year	Citations	Citation density
1	Levine, DW	A self-administered questionnaire for the assessment of severity of symptoms and functional status in carpal-tunnel syndrome	1993	1083	38.68
2	Atroshi, I	Prevalence of carpal tunnel syndrome in a general population	1999	886	40.27
3	Phalen, GS	Carpal-tunnel syndrome - 17 years experience in diagnosis and treatment of 654 hands	1966	743	13.51
4	Silverstein, BA	Occupational factors and carpal-tunnel syndrome	1987	565	16.62
5	Jablecki, CK	Literature-review of the usefulness of nerve-conduction studies and electromyography for the evaluation of patients with carpal-tunnel syndrome	1993	475	16.96
6	Gelberman, RH	The carpal-tunnel syndrome - a study of carpal canal pressures	1981	457	11.43
7	Jablecki, CK	Practice parameter: Electrodiagnostic studies in carpal tunnel syndrome - Report of the American Association of Electrodiagnostic Medicine, American Academy of Neurology, and the American Academy of Physical Medicine and Rehabilitation	2002	369	19.42
8	Rempel, D	Consensus criteria for the classification of carpal tunnel syndrome in epidemiologic studies	1998	368	16
9	Stevens, JC	Carpal-tunnel syndrome in rochester, minnesota, 1961 to 1980	1988	315	9.55
10	Dekrom, MCTFM	Carpal-tunnel syndrome - prevalence in the general-population	1992	311	10.72
11	Stevens, JC	AAEM minimonograph #26: The electrodiagnosis of carpal tunnel syndrome	1997	304	12.67
12	Kimura, J	Carpal-tunnel syndrome - localization of conduction abnormalities within the distal segment of the median nerve	1979	282	6.71
13	Padua, L	Neurophysiological classification and sensitivity in 500 carpal tunnel syndrome hands	1997	275	11.46
14	Duncan, I	Sonography in the diagnosis of carpal tunnel syndrome	1999	266	12.09
15	Buchberger, W	Carpal-tunnel syndrome - diagnosis with high-resolution sonography	1992	259	8.93
16	Altrocchi, PH	Practice parameter for carpal-tunnel syndrome	1993	252	9
17	Katz, JN	The carpal-tunnel syndrome - diagnostic utility of the history and physical-examination findings	1990	251	8.1
18	Werner, RA	Carpal tunnel syndrome: pathophysiology and clinical neurophysiology	2002	221	11.63
19	Mondelli, M	Carpal tunnel syndrome incidence in a general population	2002	220	11.58
20	Jablecki, CK	Practice parameter for electrodiagnostic studies in carpal tunnel syndrome: Summary statement	2002	219	11.53
21	Stevens, JC	Aaee minimonograph-26 - the electrodiagnosis of carpal-tunnel syndrome	1987	218	6.41
22	Beekman, R	Sonography in the diagnosis of carpal tunnel syndrome: A critical review of the literature	2003	216	12
23	Gelberman, RH	Carpal-tunnel syndrome - results of a prospective trial of steroid injection and splinting	1980	202	4.93
24	Gerritsen, AAM	Splinting vs surgery in the treatment of carpal tunnel syndrome - A randomized controlled trial	2002	201	10.58
25	Koda, Y	Switch from conventional to high-flux membrane reduces the risk of carpal tunnel syndrome and mortality of hemodialysis patients	1997	201	8.38
26	Tanzer, RC	The carpal-tunnel syndrome - a clinical and anatomical study	1959	200	3.23
27	Wong, SM	Carpal tunnel syndrome: Diagnostic usefulness of sonography	2004	199	11.71
28	Bland, JDP	A neurophysiological grading scale for carpal tunnel syndrome	2000	198	9.43
29	Klauser, Andrea S	Carpal Tunnel Syndrome Assessment with US: Value of Additional Cross-sectional Area Measurements of the Median Nerve in Patients versus Healthy Volunteers	2009	196	16.33
30	Lee, D	Diagnosis of carpal tunnel syndrome - Ultrasound versus electromyography	1999	194	8.82
31	Marshall, S	Local corticosteroid injection for carpal tunnel syndrome (Review)	2007	190	13.57
32	Dekrom, MCTFM	Risk-factors for carpal-tunnel syndrome	1990	186	6
33	Sunderland, S	Nerve lesion in carpal-tunnel syndrome	1976	183	4.07
34	Becker, J	An evaluation of gender, obesity, age and diabetes mellitus as risk factors for carpal tunnel syndrome	2002	179	9.42
35	Thomas, JE	Electrodiagnostic aspects of carpal tunnel syndrome	1967	177	3.28
36	Hobson-Webb, Lisa D	The ultrasonographic wrist-to-forearm median nerve area ratio in carpal tunnel syndrome	2008	175	13.46
37	Cannon, LJ	Personal and occupational factors associated with carpal-tunnel syndrome	1981	175	4.38
38	Geoghegan, JM	Risk factors in carpal tunnel syndrome	2004	174	10.24
39	Palmer, Keith T	Carpal tunnel syndrome and its relation to occupation: a systematic literature review	2007	169	12.07
40	Stevens, JC	Conditions associated with carpal-tunnel syndrome	1992	167	5.76
41	Armstrong, TJ	Carpal-tunnel syndrome and selected personal attributes	1979	164	3.9
42	Garfinkel, MS	Yoga-based intervention for carpal tunnel syndrome - A randomized trial	1998	163	7.09
43	Cartwright, Michael S	Evidence-based guideline: neuromuscular ultrasound for the diagnosis of carpal tunnel syndrome	2012	162	18
44	Hagberg, M	Impact of occupations and job tasks on the prevalence of carpal-tunnel syndrome	1992	162	5.59
45	Chammas, M	Dupuytrens disease, carpal-tunnel syndrome, trigger finger, and diabetes-mellitus	1995	160	6.15
46	Nakamichi, KI	Ultrasonographic measurement of median nerve cross-sectional area in idiopathic carpal tunnel syndrome: Diagnostic accuracy	2002	159	8.37
47	D’Arcy, CA	Does this patient have carpal tunnel syndrome?	2000	155	7.38
48	El Miedany	Ultrasonography versus nerve conduction study in patients with carpal tunnel syndrome: substantive or complementary tests?	2004	153	9
49	Katz, JN	Carpal tunnel syndrome.	2002	152	8
50	Kulick, MI	Long-term analysis of patients having surgical-treatment for carpal-tunnel syndrome	1986	151	4.31

A total of 27 journals published the top 50 articles. The top 10 journals are shown in Table 2. *Muscle Nerve* published most articles in CTS research (n = 8), followed by *Journal of Bone and Joint Surgery American Volume* (n = 5). *JAMA Journal of The American Medical Association* and *Neurology* published the same number of articles (n = 4). The IF of the top 10 journals ranged from 1.642 to 45.54. Only 1 journal with an IF < 2, 6 journals with an IF between 2 and 5, and 2 journals with an IF between 5 and 10. Eight journals belonged to JCR partition Q1 or Q2. It is worth to note that the *Journal of The American Medical Association* had the highest IF (45.54) in the top 10 journals.

**Table 2:** Top 10 journals of the most publications.

Rank	Journal	Publications	IF (2019)	JCR partition
1	*Muscle Nerve*	8	2.505	Clinical Neurology Q3Neurosciences Q3
2	*Journal of Bone and Joint Surgery American Volume*	5	4.578	Orthopedics Q1Surgery Q1
3	*JAMA Journal of The American Medical Association*	4	45.54	Medicine, General & Internal Q1
4	*Neurology*	4	8.77	Clinical Neurology Q1
5	*Clinical Neurophysiology*	3	3.214	Clinical Neurology Q2Neurosciences Q2
6	*American Journal of Roentgenology*	2	3.013	Radiology, Nuclear Medicine & Medical Imaging Q2
7	*Journal of Hand Surgery American Volume*	2	2.124	Orthopedics Q2Surgery Q2
8	*Journal of Occupational and Environmental Medicine*	2	1.642	Public, Environmental & Occupational Health Q3
9	*Radiology*	2	7.931	Radiology, Nuclear Medicine & Medical Imaging Q1
10	*Acta Neurologica Scandinavica*	1	2.684	Clinical Neurology Q2

**Figure 2:**
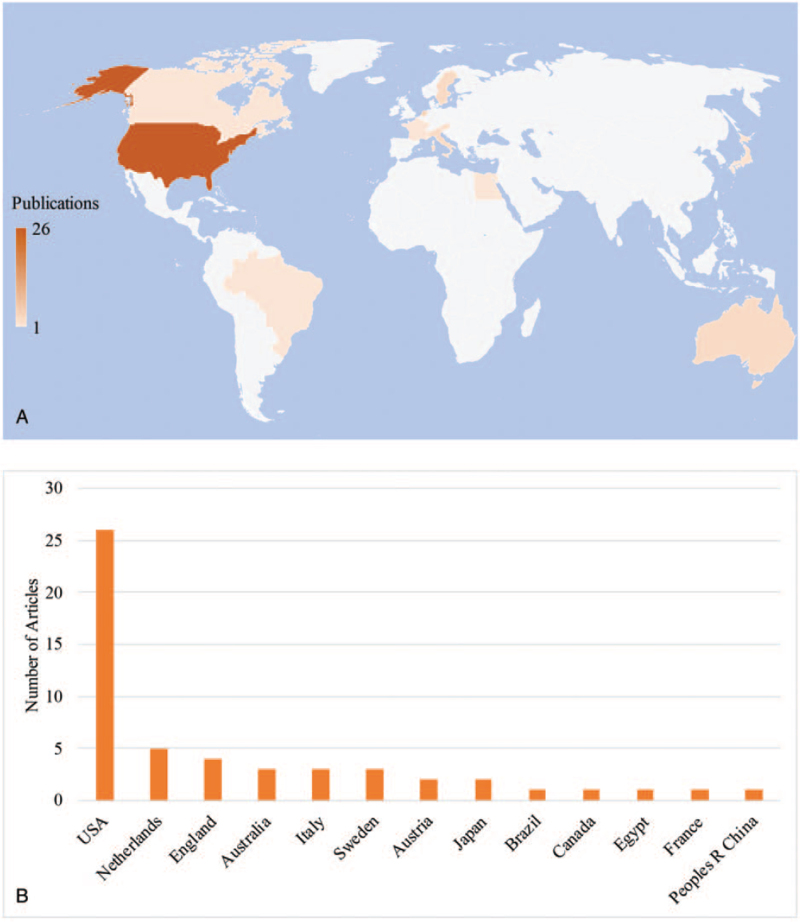
Geographical distribution of all articles. (A) The percentage of top-cited articles in different countries. (B) Publications by countries in which they were conducted.

The top 50 cited articles originated from 11 different countries (Fig. 2). The USA published the most articles (n = 26), far away from other countries. The Netherlands, which ranked second published 5 articles in the top 50, followed by England (n = 4). Brazil, Egypt, and China were the developing countries among 11 countries. Moreover, 8 countries had published at least 2 articles.

The 50 articles came from 25 institutions. The top 5 institutions with the most publications were listed in Table 3. All of the top 5 institutions are located in the USA. The top 3 institutions were University of Michigan (n = 10), University of California System (n = 8), and Mayo Clinic (n = 6).

**Table 3:** Top 5 institutions of the most publications.

Rank	Institution	Country	Publications
1	University of Michigan	USA	10
2	University of California System	USA	8
3	Mayo Clinic	USA	6
4	University of Washington	USA	6
5	Harvard University	USA	5

Our study shows that 227 authors published the top 50 cited articles. We listed the top 5 authors by publications in Table 4. Katz JN ranked first (n = 4), followed by Stevens JC (n = 3) and Andary MT (n = 3). For the *h-index* of the top 5 authors, Katz JN had the highest h-index (62), followed by Armstrong TJ (42).

**Table 4:** Top 5 Authors of the most publications.

Rank	Author	Publications	*h*-index
1	Katz JN	4	62
2	Stevens JC	3	36
3	Andary MT	3	21
4	Jablecki CK	2	17
5	Armstrong TJ	2	42

**Figure 3:**
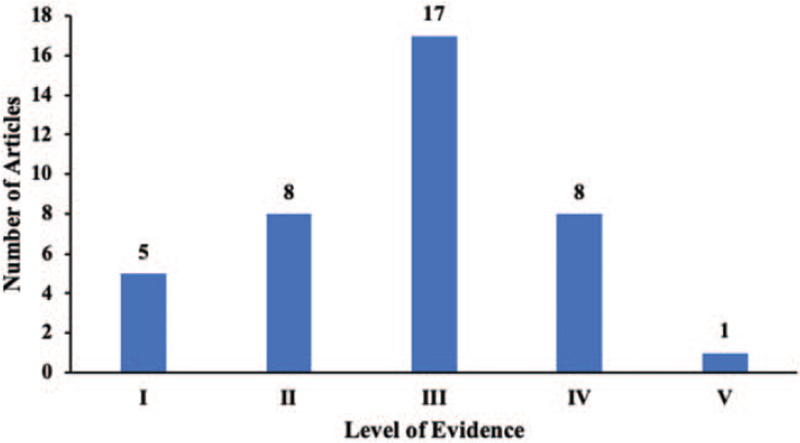
Number of clinical articles by level of evidence.

Concerning the article type and level of evidence. Most of the articles were clinical studies (n = 39), followed by review articles (n = 11), no basic science studies were found in the top 50 articles (Table 5). According to the methodology of evaluating the level of evidence, we found that most of the articles were level III evidence (n = 17). Eight articles were classified as level II and level IV evidence, respectively. In addition, 5 articles were level I evidence (Fig. 3).

**Table 5:** Study of the top 50 cited articles on carpal tunnel syndrome.

Subtype	No. of articles
Clinical practice or guideline	2
Randomized controlled trial	2
Prospective cohort study	5
Case-control study	8
Cross-sectional study	10
Case series	5
Diagnostic study	4
Expert opinion	1
Systematic review	2
Review	11

## 4 Discussion

Our study confirmed the top 50 most cited articles pertaining to CTS published between 1959 and 2012. These top-cited articles might be the most representative and influential works in this field. The majority of the most cited articles were published in the journal titled *Muscle Nerve*. The most productive country is the USA, far away from other countries. The University of Michigan was the most active institution, which also came from the USA. In addition, Katz JN with the highest *h-index*, published 4 papers within the 50 papers. The type of the top 50 cited articles were clinical studies and reviews. Most of the clinical studies were level III evidence. Researchers can understand the essential contents of the CTS research through our findings.

The most cited article by Levine et al^[19]^ in 1993 received 1083 citation times and 38.68 citation density. The authors developed a self-administered questionnaire with the characterize of reproducibility, internally consistency, and responsiveness to clinical change. The questionnaire, which contains 2 scales, could be applied to supplement other variables measured in the CTS research field. Despite the fact that many scales have been applied to evaluate upper limb function,^[20,21]^ it is certain that the Boston Carpal Tunnel questionnaire by Levine is the most widely used instrument for patients with CTS.^[22]^ Thus, the top 1 article has a strong impact in this field.

One article with the highest citation density (40.27) by Atroshi et al^[23]^ in 1999 ranked second in the top 50 cited articles. The authors conducted a study among 3000 participants to estimate the prevalence of CTS in southern Sweden. The sample size of the study was large and had a high response rate. They concluded that the prevalence of CTS is 14.4% in the general population, and the prevalence ratio of females and males is 1.4. They also found that obesity might be a risk factor for CTS. Besides, blue-collars are more likely to suffer from CTS than white collars.

All the 50 cited articles were published in 27 different journals. From the JCR partition results in the top 10 articles, we can consider that researchers from different fields focus on CTS research, such as neurology, orthopedic, and radiology. Besides, IF is not the only indicator to evaluate the quality of journals. We should also combine it with JCR partition.^[24]^ Although the IF of *Journal of Hand Surgery American Volume* is relatively low (IF = 2.124), the JCR partition is Q2 in the orthopedics and surgery field. It might still be a vital journal in the specialized field. Most journals belong to Q1 or Q2 zone in a different area, except *Muscle Nerve* and the *Journal of Occupational and Environmental Medicine*. *JAMA Journal of The American Medical Association* with the highest IF (45.54) published 1 paper with the highest citation density (40.27), and the JCR partition is Q1. It is enough to identify that this journal is of great significance.

The USA was far from other countries in some fields, such as spine tumor,^[25]^ rheumatology,^[26]^ and rotator cuff research.^[27]^ Our results also identified that the USA had a substantial influence on CTS research. We found that the USA published more than half of the top 50 most-cited articles, and all the top 5 institutions were originated from the USA. This might be associated with the strong comprehensive economic strength of the USA. Katz JN with the highest *h-index* was from Boston. He was committed to carpal tunnel release surgery.^[28,29]^ Furthermore, he made a clinical practice about CTS was published in *The New England Journal of Medicine* (IF = 74.699) in 2002.^[30]^ The clinical practice proposed how to diagnose, assess, and treat CTS patients based on other guidelines.

We found that most of the top 50 articles had a lower level of evidence (III, IV, or V), which is consistent with other bibliometric studies.^[31,32]^ There is no basic science in the top 50 articles of CTS, indicating that most researchers prefer to cite clinical trials.^[33]^ Level III evidence was the most common level of evidence in the top 50 articles. This might indicate that the citation times were not influenced by the level of evidence.^[34]^ In addition, only 2 RCTs ranked in the top 50 articles, more high-level trials related to CTS are needed in the future.

There are some limitations in our study. First, citation numbers could be influenced by external factors, bias, and controversy.^[35]^ Thus, ranking literature by citation times might not be comprehensive. Second, we only searched the data from the WoS, some high-level articles pertaining to CTS might be omitted. Third, textbooks, lectures, and conference abstracts did not constitute part of our filter criteria.

## 5 Conclusion

This study identified the top 50 articles related to CTS. These articles might help researchers realize the research foundation in this field, especially some critical literature. In addition, we found that the USA was still the leading country, and Katz JN with the highest *h-index* is an influential researcher in this field. Katz JN might be a potential collaborator in this field. The majority of the most influential articles were level III evidence. Researchers should commit to high-level articles in the future.

## Author contributions

**Conceptualization:** Lei Lei, Moxian Chen.

**Funding acquisition:** Moxian Chen.

**Investigation:** Ya Zhou.

**Methodology:** Shanshan Yin.

**Project administration:** Lei Lei, Moxian Chen.

**Resources:** Xuan Xu.

**Supervision:** Rui Xu.

**Writing – original draft:** Lei Lei, Shanshan Yin.

**Writing – review & editing:** Fanyuan Meng, Lijuan Ao.
